# Alzheimer’s Disease: An Overview of Major Hypotheses and Therapeutic Options in Nanotechnology

**DOI:** 10.3390/nano11010059

**Published:** 2020-12-29

**Authors:** Mugdha Agarwal, Mohammad Rizwan Alam, Mohd Kabir Haider, Md. Zubbair Malik, Dae-Kwang Kim

**Affiliations:** 1 Department of Biotechnology, Jaypee Institute of Information Technology, Noida 201309, India; agarwalmugdha1@gmail.com; 2 Department of Medical Genetics, School of Medicine, Keimyung University, Daegu 42601, Korea; mdrizwan2001@gmail.com; 3 Vellore Institute of Technology, Vellore 600127, India; kabirhaider2000@gmail.com; 4 School of Computational and Integrative Sciences, Jawaharlal Nehru University, New Delhi 110067, India; 5 Hanvit Institute for Medical Genetics, Daegu 42601, Korea

**Keywords:** Alzheimer’s disease, neurodegeneration, blood–brain barrier, nanotechnological approaches, drug delivery

## Abstract

Alzheimer’s disease (AD), a progressively fatal neurodegenerative disorder, is the most prominent form of dementia found today. Patients suffering from Alzheimer’s begin to show the signs and symptoms, like decline in memory and cognition, long after the cellular damage has been initiated in their brain. There are several hypothesis for the neurodegeneration process; however, the lack of availability of in vivo models makes the recapitulation of AD in humans impossible. Moreover, the drugs currently available in the market serve to alleviate the symptoms and there is no cure for the disease. There have been two major hurdles in the process of finding the same—the inefficiency in cracking the complexity of the disease pathogenesis and the inefficiency in delivery of drugs targeted for AD. This review discusses the different drugs that have been designed over the recent years and the drug delivery options in the field of nanotechnology that have been found most feasible in surpassing the blood–brain barrier (BBB) and reaching the brain.

## 1. Introduction

The most prevalent cause of dementia is Alzheimer’s disease (AD), a condition that affects approximately 50 million people worldwide, and the case of dementia is estimated to reach 131.5 million by the year 2050 [1]. AD is characterized by cognitive decline, behavioral change and inability to perform daily life activity [2,3]. Lack of successful Aβ clearance are thought to cause the onset or development of AD in most situations [4,5,6,7]. Available drugs that lower Aβ has been ineffective in preventing cognitive decline [8,9,10]. Despite continuous efforts by researchers towards finding a cure for the disease, more than a century since AD was first discovered, we have still been unable to come up with any significant treatment option, owing mainly to the lack of efficient drug delivery methods and several loopholes in the conventional drugs focusing on the symptomatic management of the disease and these drugs unlikely to stop the disease development [11,12,13].

Currently the FDA-approved drugs for AD in the market have limitations like high dosage regimes, low bioavailability, gastrointestinal tract side effects and ineffectual brain targeting, which ultimately lead to incompliance with the patient and discontinuation of the treatment [14,15]. This is where the role of nanotechnology comes into play. Advancements in this field have given rise to ease in the delivery of therapeutic molecules across the BBB and reaching the central nervous system (CNS) [16], along with the removal of other aforementioned impediments in the treatment process of AD.

## 2. Pathophysiology of the Disease

Alzheimer’s is characterized by the presence of amyloid beta plaques and neurofibrillary tangles that are formed in the patient’s brain [17]. Since the disease’s pathogenesis is multifactorial, the detection of behavioral and memory changes is difficult [18,19]. The mutations in three major genes encoding—amyloid precursor protein (APP) on chromosome 21, Presenilin-1 (PS1) on chromosome 14 and Presenilin-2 (PS2) are reported to be responsible for the formation of the same [20]. The mutations in these genes lead amyloid-β protein (Aβ) to form senile plaques in the extracellular region and the hyper phosphorylation of Tau protein that forms the neurofibrillary tangles intracellularly [21]. This causes widespread damage to nerve cells throughout the brain cortex, accompanied by early loss of cholinergic neurons from the basal region of the forebrain. There are a number of hypotheses that aid in the therapeutic formulation for AD and that have been discussed before. Some pharmacological treatments available for Alzheimer’s disease are shown in Table 1.

### 2.1. The Amyloid-Beta Hypothesis

This hypothesis is the most recognized one amongst researchers, owing to its explanation for the senile plaque formation and the accumulation of Aβ oligomers as the major highlight of the disease [22]. The proteolysis of transmembrane protein APP by beta and gamma secretases forms single units of Aβ, which further undergo certain structural modifications to form sheets of oligomers that are harmful in nature. These oligomeric sheets aggregate to form plaques and tangles. The Aβ protein has two subunits—Aβ40 and Aβ42, where the latter is soluble. The APP is normally cleared by an enzyme called alpha secretase, which yields sAPP-alpha [23,24,25]. The sAPP-alpha is responsible for memory and learning activities of the brain, fighting against stress conditions and in maintaining neuronal excitability. In the diseased condition, the APP is cleaved by beta secretase into sAPP-beta and C99 fraction, which is membrane bound. Gamma secretase acts upon the C99 fraction producing either Aβ40 or Aβ42, which cause the plaques to deposit [26,27,28]. This disrupts the normal functioning of sAPP, leading to metabolic changes, decreased neuronal excitability, conditions favoring oxidative stress and dysregulated calcium homeostasis.

**Figure 1:**
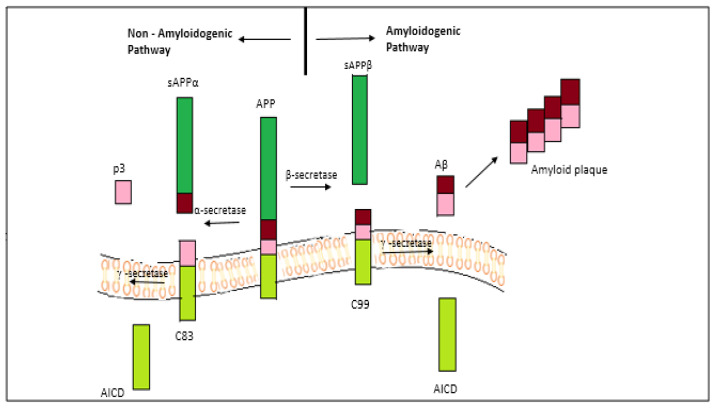
Amyloid precursor protein (APP) cleavage in normal (non-amyloidogenic) and AD (amyloidogenic) pathways.

Recently, it has been discovered that APP cleavage occurs by a third way involving η-secretase [29]. The η-secretase is found to cleave APP at amino acids 504–505, which generates carboxy-terminal fragments Aη-α and Aη-β of higher molecular mass after undergoing a second cleavage by α-and β-secretase, respectively. An Aβ (1–16) fragment is contained by the Aη-α sequence, which is found to be neurotoxic. Aβ plays a role in memory and synaptic plasticity, although its proper function in the brain remains unknown yet [30]. AD has two main forms: A late-onset form known as sporadic AD, which is more common; and an early-onset or familial form with 5% of all AD cases [31]. It has been seen that in individuals suffering with Down’s syndrome (or trisomy 21), there is an increased risk of familial AD, as they are carriers of an extra chromosome 21 where the gene responsible for the formation of APP is present (Figure 1).

Similarly, a mutation in some of the many genes including PSEN1 and PSEN2, which code for APP, Presenilin1 and Presenilin2 which are also the two subunits of γ-secretase, have been recognized as the causative genes for familial AD [20]. These mutations cause an enhanced production of Aβ, with the mutations on PSEN1 particularly leading to an increase in formation of Aβ (1–42). The apolipoprotein (ApoE), which is involved in the clearance of Aβ, is a major genetic risk factor associated with late-onset AD [32,33]. There are three categories in which the mutations have been divided: The N-term mutation occurring at the cleavage site for β-secretase, the C-term mutation at the cleavage site for γ-secretase, and the mutation occurring in the mid-domain Aβ region. The mutations that occur at γ-secretase cleavage site can increase the ratio of Aβ1–42/Aβ1–40 and alter the position of cleavage. There is an increase in the rate of proteolysis of APP by β-secretase due to the mutations at β-secretase cleavage site. While the mutations occurring at the mid-domain of Aβ region in APP lead to an increase in the Aβ propensity for formation of oligomers and fibrils that disrupt the Aβ assembly. Many studies have reported mutation at the γ-secretase processing site of APP [34,35,36,37,38]. More than the protofibrils and fibrils, it is the oligomers that are found to be more toxic for the brain cells (Figure 1). This is because the oligomers are capable of permeating the cellular membranes causing cellular dysfunction and death.

The cascade of Aβ involves a number of factors and modulators that have an essential role each to play. Metal ions such as iron, zinc and copper are found to be present in the amyloid plaques and are involved in creating conditions of oxidative stress, as well as in the modulation of aggregation process by binding to Aβ [39]. These ions function by acting on the kinetics or thermodynamics to affect the structural morphology of the aggregates formed. The amyloid aggregates that have metal ions entrapped within them have been found to be highly toxic as they can cause the production of reactive oxygen species (ROS) which have a deleterious effect on both the Aβ peptide and the biomolecules in the vicinity [40]. Release of inflammatory factors like reactive oxygen species (ROS), nitric oxide synthase (NOS) and prostaglandins is stimulated bringing about the death of nerve cells [41].

There are drugs that serve as beta and gamma secretase inhibitors, including Elenbecestat (E2609), verubecestat (MK-8931) and Semagacestat [42], but none of them have cleared all the steps of clinical trials [43]. Similarly, beta secretase modulators also failed due to their unsafe use to patients. The cleavage of APP by α- and γ-secretase produces sAPPα (soluble amino terminal ectodomain of APP), a larger C83 fragment (carboxy terminal) and a smaller fragment p3. This pathway does not give rise to amyloid beta (Aβ) production. The cleavage of APP by β-secretase (BACE1) and γ-secretase produces sAPPβ, C99, AICD (APP intracellular domain) and leads to the formation of Aβ [44,45].

### 2.2. The Tau Hypothesis

**Figure 2:**
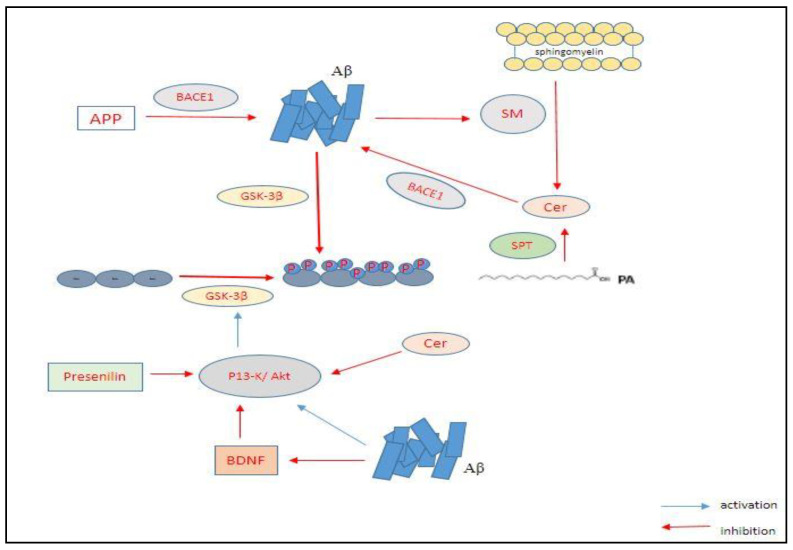
Interactions occurring between amyloid beta (Aβ), hyperphosphorylated tau, glycogen synthase kinase 3β (GSK-3β) and ceramides (cer).

Tau is present in axons and dendrites and it regulate microtubules function [46,47,48,49]. The biological functioning of Tau is regulated by the level of its phosphorylation in the brain. Tau generally contain 2–3 mole of phosphates per mole of protein, but, in the case of AD brain, it contains more phosphates [50,51]. An excessive or hyper phosphorylation of microtubule-associated protein, Tau in case of AD, leads to its transformation from normal adult Tau to a paired helical filament (PHF-tau) of it, impairing its ability to bind to the microtubules stably [52,53]. This is a result of mutations that cause tau to aggregate and attain an insoluble structure, as opposed to their normal soluble structure. The insoluble state leads to enormous destruction of cytoplasmic functions of the nerve cells and a disruption in axonal transport, ultimately leading to dementia and neuronal death [54]. Neuronal cell death mediated by tau along with hyperphosphorylation also requires the activation of glycogen synthase kinase 3β (GSK3-β). Previous studies have reported that inhibition of GSK3-β decreases tau phosphorylation [55,56] (Figure 2). The tau pathology states that the formation of neurofibrillary tangle (NFT) spreads to various parts of the brain by following a stereotyped pattern of six pathological stages, wherein the first two stages the cognition of the patients is impaired.

There is a neurodegenerative “triad” of cellular changes that has been revealed via the microscopic analysis of different models of AD animals as well as AD patients, which affects the disease development. This triad comprises of: (a) A density decrease accompanied by a change of shape of the dendritic spines, which are the postsynaptic excitatory input site of most neurons; (b) neuronal cell loss in specific regions of the brain; and (c) a subset of neurons that undergo dendritic simplification. Over the years, the emergence of different events of the neurodegenerative triad may occur at different points of time with the progression of AD [57]. Studies on organotypic cultures and animal models have shown that the loss of dendritic spines and changes in synapse begin to surface very early in the disease. These changes; however, can be reversed if the amount of Aβ is reduced and the cAMP/PKA/CREB signaling pathways are restored [58] (Figure 2).

Loss of neurons and dendritic simplification are events that are found to appear later in the disease, which suggests that the aspects of neurodegenerative triad dependent on tau are characteristic of further disease progression. It is clear that the loss of neurons is an irreversible event, the reversibility of dendritic simplification; however, is yet to be established. Since it is known that the loss of synapse and dendritic simplification is caused due to a disruption of the cytoskeleton, drugs capable of modulating dynamics of the cytoskeleton, and the microtubular network dynamicity in particular, can serve as therapeutic options for the counteraction of tau-mediated changes [59]. In tau knockout animal models, it was observed that there was no major effect on the development and function of brain. This led to an increased interest in the development of such strategies that were directed to tau, as they would have fewer side effects as compared to the drugs that were directed on APP and Aβ, which are involved in numerous biological processes. There are six isoforms of tau present in the CNS that are produced by the alternative splicing of three axons [60,61]. Any error in the splicing of tau, particularly an increased formation of longer isoforms of tau can lead to tauopathies. PHF-tau is phosphorylated at its serine and threonine residues several times [62,63,64,65].

Tau undergoes a number of post-translational modifications such as ubiquitination, acetylation, methylation and O-glycosylation. Studies on mouse models have shown that tau turnover was reduced and tau aggregation was increased through the acetylation of tau at Lys174, which was identified as an early modification in the brains of AD patients [66]. A number of interaction partners of tau that could be of functional importance have been found apart from microtubules, such as annexin A2, a membrane associated protein contributing to the axonal localization of tau; fyn, a non-receptor tyrosine kinase of the src-family involved in post-synaptic Aβ toxicity; and a primary tau phosphatase, protein phosphatase 2A.

GSK-3β is the major kinase involved the phosphorylation process of tau. With the aid of GSK-3β, the intracellular aggregation of Aβ occurs that might also contribute to the hyperphosphorylation of tau. Additionally, the Aβ aggregation acts on sphingomyelinases (SM; enzymes involved in the degradation of sphingomyelin) affecting ceramide production. The ceramides produced act on β-secretase (enzyme involved in proteolytic cleavage of APP) leading to increased Aβ production. Presenilin and brain-derived neurotrophic factor (BDNF) are responsible for modulating these interactions by the P13-K/Akt signaling pathway. P13-K causes activation of the Akt/protein kinase B, which further causes phosphorylation of GSK-3β inducing its inactivation and; thus, downregulating phosphorylation of tau. 

### 2.3. The Cholinergic Hypothesis

It is the oldest known hypothesis which forms the basis of most of the drugs available in the market today [67]. According to this hypothesis, there is a reduced rate of production and transportation of the neurotransmitter acetylcholine in the brains of AD-affected individuals [68]. This neurotransmitter is used by all the cholinergic nerve cells and has an important role in the peripheral and central nervous systems, as it is used by all pre and post-ganglionic parasympathetic nerve cells and also all the pre-ganglionic sympathetic nerve cells. Studies have shown that the cholinergic system is a crucial contributor to the learning and memory processes [69,70,71,72,73]. In AD, the cholinergic neurons forming the nucleus basalis of Meynert are specifically degenerated, which causes memory loss seen in the AD patients [74,75,76,77,78]. The nucleus basalis region of a healthy adult brain contains about 500,000 cholinergic neurons, whereas a mere 100,000 remain in advanced AD patients [79]. There is a major decrease in the transcription of enzyme choline acetyltransferase (ChAT) in the remaining cholinergic nerve cells, leading to diminished activity of ChAT and the condition of dementia. It has also been found that the release of ACh in the forebrain can be regulated by stress conditions. A disruption in its transmission process is capable of affecting all aspects of cognition, the cortical and hippocampal information processing and behavior. Any change from the normal in the cholinergic inputs to the brain cortex leads to an impairment in attention and cognitive functions such as the processing of instructions required for decision making.

Moreover, it has been found that memory and knowledge encoding is impaired upon the blockage of CA3 cholinergic receptors. A reduction in the cholinergic neurons and the resulting impaired dopaminergic transmission has also been considered as a major factor related to psychiatric symptoms in AD. This hypothesis can be supported by the fact that there is an increase in the efflux of dopamine in nucleus accumbens as seen in M4 knockout mice. The loss of cholinergic neurons is not only found in the case of AD, but also in a number of other neurodegenerative disorders including PD, HD and ALS where a significant decrease in the activity of ChAT is seen [80]. The cholinergic synapses are severely affected by Aβ, which can be correlated to the cognitive decline. The hippocampal synaptic transmission is changed with respect to changes in the expression of synaptophysin, a major presynaptic vesicle protein p38, which correlates highly with the neuropathology and memory loss observed in AD patients. A severe deficit in basal synaptic transmission (~40%) was recorded upon electrophysiological studies in the hippocampal region of mutant APP mice.

**Figure 3:**
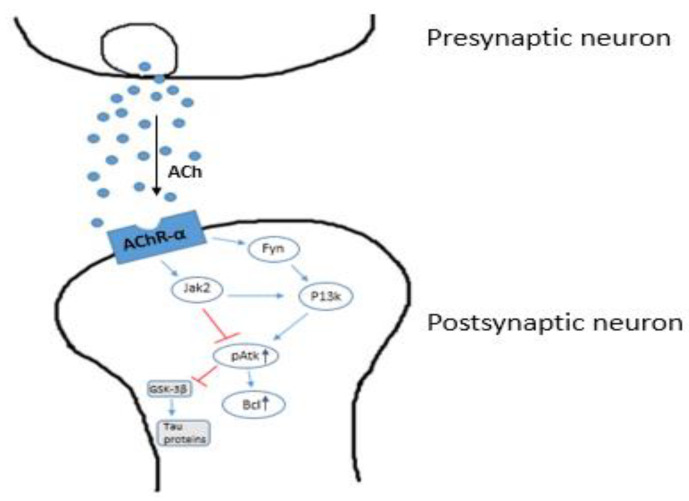
A diagrammatic representation of the neuroprotective activity of Acetylcholine esterase (AChE) inhibitors (such as Donepezil and Galantamine).

Cholinergic neurons play a significant role in promoting memory and cognitive functions, as proven via experimentation studies on rat models using cholinergic antagonists which showed cognitive damage in the rats [81,82]. The coupling of M1 muscarinic receptors to G-proteins is damaged in the neocortex of AD patients. It has been demonstrated that the extent of this uncoupling of M1 and G-protein is linked to the graveness of cognitive symptoms in AD. Further, a shift in the processing of APP towards the non-amyloidogenic pathway occurs when muscarinic receptors are activated. M1 receptor signaling is also known to be affecting a number of hallmarks in AD, such as cholinergic deficiency, Aβ and tau pathologies and cognitive dysfunctions. M1 receptor activation can activate PKC and inhibit GSK3-β, which can lead to a significant reduction in tau hyperphosphorylation. AF267B, a known M1 agonist, is capable of rescuing the decline in cognition via a decrease in Aβ42 and abnormalities associated with tau in the cortex and hippocampus, as seen in an AD mouse model. These findings and several other studies have produced the option of M1 acetylcholine receptor agonists as potential therapeutic tools for treating AD. Acetylcholinesterase (AChE) inhibitors, like Donepezil, work by decreasing the hydrolysis of Ach and improving memory and cognition [83], while Rivastigmine serves an additional function of blocking not only acetylcholinesterase, but also butyl cholinesterase to increase the chances of managing AD (Figure 3). Similarly, there is Galantamine which is an effective drug working as an AChE inhibitor on the same mechanism.

They stimulate nAchr (nicotinic acetylcholinesterase receptor) through a site other than the AChE binding site under normal condition. α7 nAChR, when stimulated by these drugs, causes the activation of PI3K (phosphatidylinositol 3-kinase) due to the activation and association of Jak2 (janus activated kinase 2) with the non-receptor type tyrosine kinase Fyn. Activation of PI3K activates Akt by phosphorylation (Akt-p). Nicotine treatment increases the level of Akt-p. It further increases the Bcl-2 expression level, preventing the death of nerve cells. The hypoactivation of α7 nAChR decreases activation of PI3K and Jak2. This increases GSK-3β enzyme activity, which increases phosphorylation of tau proteins causing neuronal death [84].

### 2.4. The Dendritic Hypothesis

This hypothesis focusses on the degeneration of dendrites accompanied with their structural and functional disturbances caused in AD. The activation of N-methyl-d-aspartate receptor (NMDAR) finds a major implication in Alzheimer’s disease, which has been studied extensively over the last few years. NMDARs are crucial for the processes of neurotransmission and synaptic plasticity in the brain [85,86,87,88]. Glutamate is the most abundantly present excitatory neurotransmitter found in the mammalian CNS. Ligand-gated ionotropic glutamate receptors (iGluRs) play a pivotal role in the excitatory neurotransmission, and a disruption in their normal signaling process is associated with a number of neuropathological diseases like AD, PD, HD and multiple sclerosis, which makes them important therapeutic drug targets [89,90]. There are three subfamilies of iGluRs, which are: a-amino-3-hydroxy-5-methyl-4-isoxasolepropionic acid receptors (AMPARs), kainate receptors and NMDARs. Owing to certain unique properties associated with it, the NMDAR is distinct from the other two iGluRs in the voltage-dependent activation [90,91]. The NMDAR has high permeability to calcium ions (Ca2+) and its ligand-gated kinetics is relatively slow, which makes it crucial in synaptic functions. The Ca2+ channel of NMDAR remains blocked by Mg2+ at the resting membrane potential (−70 mV), while this blockade is removed during the long term potentiation (LTP), allowing a prolonged and strong release of glutamate from the presynaptic terminal [92,93]. This leads to the activation of AMPARs and subsequently the depolarization causes Mg2+ removal from the NMDAR channel and a Ca2+ influx. This also triggers the activation of a Ca2+/calmodulin-dependent protein kinase II (CaMKII)-mediated signaling cascade, which causes an increase in the synaptic strength. A moderate activation of NMDARs causes a moderated increase in the postsynaptic Ca2+ and a trigger of phosphatases mediating long term depression (LTD) [94].

There are two types of membrane NMDARs: Synaptic and extrasynaptic. By the activation Ca2+ dependent transcription factors, such as cyclic-AMP response element binding protein (CREB) and the suppression of apoptotic pathway and caspases, the synaptic NMDAR helps in promoting the expression of survival gene [95]. The extrasynaptic NMDAR, on the contrary, is involved in glutamate excitotoxicity and cell death. Its responses are strongly linked with the physiological changes in AD. The activation of the two NMDARs occurs by two different endogenous coagonists: D-serine for synaptic NMDAR and glycine for extrasynaptic NMDAR. The signaling pathway mediated by extrasynaptic NMDAR is known to antagonize the cell survival pathway via CREB inactivation and FOXO (forkhead box) transcription factor activation which promotes pro-apoptotic and oxidative stress signaling [96]. AD affects the NMDAR coagonist levels. As the binding of coagonist D-serine or glycine is needed for the complete activation of NMDARs by glutamate, the coagonists serve an essential modulatory role in the functioning of NMDAR. One of the FDA approved drugs for AD, memantine, works as an NMDAR antagonist and targets against the extrasynaptic NMDAR. The level of Ca2+ entering through NMDAR that exceeds the pathological normal [97] determines the level of toxicity produced. It results in a gradual loss of synaptic plasticity and neuronal death eventually that can be clinically correlated to a decline in memory and cognition in AD patients. Not only is the electrophysiological functioning of NMDARs directly modulated by Aβ, an elevation in the levels of synaptic currents and collateral toxicity mediated by NMDARs is also brought about by Aβ in AD. NMDAR antagonists such as MK-801 serve as the blockers or attenuators of the same.

Since NMDARs also play an essential role in cell survival, a balance in their level of signaling is of utmost importance, such that it is sufficient for neuronal survival and at the same time, does not bring about neurodegeneration as in the case of AD [98]. The amount of glutamate available for signaling depends upon its uptake and recycling system, which was found to be severely compromised in AD. A study on an AD patient revealed that there is severe reduction in the capacity of glutamate transporter and protein expression [99]. The expression of presynaptic proteins, including syntaxin and synaptotagmin, which comprise the neurotransmitter release machinery, are known to be greatly reduced due to Aβ. Aβ can interact with NMDARs indirectly via such synaptic proteins as PSD95 [100]. The deficiency in presynaptic proteins leads to a compromised availability of glutamate, thus producing excitotoxicity, an effect often seen in degenerating nerve cells. The activation of N-methyl-D-aspartate (NMDA) receptor by the amyloid beta and prion proteins, in addition to the activation of Fyn by prion protein and Fyn tyrosine kinase-metabotropic glutamate receptor 5 complex (FynmGluR5), results in the decrease of NMDA receptors [101]. Fyn, upon overstimulation, causes cognitive damage and synaptic losses leading to the disease condition [28]. Memantine, a drug that functions as an NMDA receptor blocker, has been approved by FDA and is currently in use, although relatively preferred less in comparison to AChE inhibitor drugs.

#### 2.4.1. Wnt/Beta-Catenin Signaling

The low density lipoprotein receptor-related protein 6 (LRP6) is a major Wnt co-receptor required to activate the Wnt/β-catenin pathway on the cell surface. LRP6 is strongly related to the signaling pathway of glucose and lipid metabolism [102,103,104]. The Wnt/β-catenin pathway is responsible for the regulation of a number of significant cellular functions, such as cell growth and proliferation, differentiation and migration. A dysregulation in this pathway has been found to play an important role in AD pathogenesis. The pathway is activated when Wnt proteins bind to the Frizzled (Fzd) receptor family’s cysteine rich domain and Wnt co-receptor LRP6. Studies have shown that susceptibility of neurons to death induced by amyloid beta increases with a decrease in the Wnt/β-catenin signaling, whereas the same Aβ-induced neuronal death can be prevented by the activation of this signaling [105,106]. Whether there is occurrence of neurogenesis in an adult human brain has been a much debated topic. There is evidence supporting the occurrence of neurogenesis in the hippocampal region of the human brain which experiences a sharp decline in the case of AD.

Studies show that the Wnt/β-catenin signaling has a key role in the regulation of neurogenesis in adult hippocampus, as it is activated by the Wnt7a gene at multiple steps of neurogenesis along with other specific genes controlling the neuronal cell cycle and differentiation processes [105,107]. It was found that, in aged mice, the Wnt proteins secreted by astrocytes decrease, causing decreased Wnt/β-catenin signaling, decrease in the level of survivin (responsible for mitotic regulation) in neural progenitor cells (NPC) and an impaired neurogenesis [108]. The activation of the Wnt/β-catenin signaling pathway determines the activation of survivin and transcription factors, such as NeuroD1 and Prox1, which are involved in the generation of hippocampal granule cells [109]. Furthermore, this signaling pathway plays an essential role in maintaining the synaptic plasticity. The Wnt proteins are involved in synapse formation and the pre- and post-synaptic modulation of neurotransmission. LRP6 helps in the in vivo and in vitro development of excitatory synapse and its deficiency leads to abnormal synapse and cognition, as found in aged mice models [107,110]. Other functions associated with the activation of LRP6-mediated Wnt/β-catenin pathway include the function and formation of blood–brain barrier (BBB), by activating the signaling in endothelial cells of the BBB, and inhibition of β-plaque formation via inhibition of transcriptional expression of β-site APP cleaving enzyme (BACE1) [111,112,113,114]. Interaction of LRP6 with APP lowers the production Aβ and the suppression of tau phosphorylation via suppression of the GSK3β kinase activity.

#### 2.4.2. GSK3-β Activity

Glycogen synthase kinase-3 (GSK-3) is a serine-threonine kinase that functions as a key regulator in many biological pathways, some of which have their implications in AD. It has two isoforms, GSK3-α and GSK3-β, each encoded by a different gene. The GSK3-β is found in abundance in the CNS, with the level of its expression increasing with age, and its activity superseding the normal in case of AD patients [56]. The over activation of this kinase is linked with the deposition of amyloid beta, memory impairment and plaque-related inflammatory responses mediated by microglia. The cleavage of APP in the non-amyloidogenic pathway that involves α- and γ-secretases has three members ofthe α-disintegrin and metalloproteinase (ADAM) family (ADAM-10, ADAM-17, and ADAM-9) forming the α-secretase complex [113,115]. GSK3-β is known to inhibit the activity of ADAM and thus downregulate the activity of α-secretase complex. Amongst the proteins constituting the γ-secretase complex, the function of presenilin (PSEN) 1 is affected by GSK3-β [116]. Since APP and PSEN1 are both substrates of GSK3-β, it interferes with the production of Aβ at the step of APP cleavage by γ-secretase [117].

The signaling of this kinase is found to be activated by Aβ, as its inhibition via phosphorylation is prevented by Aβ in transgenic AD models of animals. Similarly, an increased GSK3-β activity was observed in the brains of AD patients. A reduction in Aβ production, as well as Aβ-induced neuronal toxicity, was seen upon the inhibition of GSK3-β in mice models of AD. BACE1 mediates the APP cleavage by NF-kB signaling mechanism [112,113,114]. The expression of BACE1, which is found to be increased in AD patients, can be downregulated upon GSK3-β inhibition [112]. GSK3-β is known to phosphorylate at least 36 different residues in the tau protein, with the major sites identified to be Ser199, Thr231, Ser396, Ser413 and other sites of moderate phosphorylation including Ser46 and Ser202/Thr205 [118,119,120]. Apart from GSK3-β, CDK-5 and PKA are two other kinases associated with microtubules and tau protein. Tau is a microtubule-associated protein (MAP) that functions as a regulator of microtubule formation and its stability [121,122]. A combined action of GSK3-β and CDK-5 is required for the formation of paired helical filaments of tau (PHF tau) [123]. This form of the tau protein is insoluble and can aggregate and deposit inside the nerve cells leading to the formation of neurofibrillary tangles (NFTs). The PHF tau is unaffected by the action of proteases or phosphatases. Studies have shown that GSK3-β is activated by an elevation in oxidative stress, neuroinflammation and apoptotic cell death that is brought about by hyperphosphorylated tau [117]. Along with neuronal death and hyperphosphorylation in tau, GSK3-β overexpression has been found to cause a failure in mice to perform the Morris water maize test [124]. It causes increased apoptosis in some particular areas of the brain, including the hippocampus which controls memory and cognition and is severely affected in AD. However, it was seen that these effects were reversed and tau hyperphosphorylation was reduced upon restoration of GSK3-β to the normal levels. GSK3-β can regulate the stability of axons directly by interacting with microtubules, owing to its capacity to phosphorylate numerous MAPs [125].

The MAP-2 and tau phosphorylated by GSK3-β are deprived of their affinity towards microtubules, making them unstable in nature. Failure in axonal transport results, which adds significantly to the pathology of AD [125]. GSK3-β is known to be involved in the metabolism of choline and regulation of choline acetyltransferase (ChAT), as well as acetylcholinesterase [126]. A reduction in phosphorylation of Ser9 of GSK3-β has shown to cause a loss of cholinergic nerve cells from the basal forebrain and hippocampal area, and an enhanced phosphorylation of tau [118,119,120]. GSK3-β plays an important role in the process of inflammation, as it can regulate the process in a positive manner by promoting the activity of pro-inflammatory cytokines [127], while lowering anti inflammatory cytokines activity. Over the recent years, a number of GSK3-β inhibitors have been developed, both ATP-and non-ATP-competitive types [128]. Since the non-ATP-competitive GSK3-β inhibitors prove to be more sensitive, selective and less toxic in nature, they are preferred more than the ATP-competitive type (Indirubin).

### 2.5. The 5-HT_6_ Receptor Hypothesis

It has been found in recent studies that the inhibition of antagonists at serotonin type 6 (5-HT_6_) receptor can improve cognition in AD [129,130,131,132]. The injection of 5-HT_6_ receptor antagonists in rodent models led to significant cognitive improvement [133,134]. This receptor is also found to be involved in amyloid protein formation and the signaling of Fyn [135]. These receptors can; therefore, play a major role in AD treatment as the inhibitors can also stop Fyn activation and deposition of amyloid. All the current drugs, apart from AChE inhibitors, have adverse effects associated with them which fails them in phase 2 or 3 of clinical trials [136]. It is worth noting that Dimebon (latrepirdine, also known as Dimebolin) was initially developed as an antihistamine drug. For 5-HT_6_ receptors (ki = 34 nM), this compound shows strong affinity. After a very promising phase 2 review, Dimebon gained widespread attention as a possible treatment for AD [137]. A more recent multinational phase 3 research; however, has shown no changes [138].

## 3. Nanotechnology-Assisted Drug Delivery Strategies for AD

All the drugs currently approved for the treatment of AD are available as oral formulations, barring Rivastigmine which also has a transdermal patch available [28,139]. Since the drugs need to reach the CNS in order to control the progression of disease or its symptoms, a much higher dose needs to be consumed because of the large fraction of drug that is lost along the way in GI tract and metabolism in the hepatic region [28,139]. Furthermore, the drug needs to bind to serum albumin in the blood stream in order to sustain a decent half-life, before it finally reaches the BBB [140,141]. Consuming these dosages leads to the patients suffering with side effects like nausea and diarrhea and reducing their compatibility. Nanotechnological advancements in the recent years have provided us with the option of nanoparticles that help in overcoming these hurdles in drug delivery, particularly in improving the side effects by reducing the dosage and in easily traversing across the BBB to provide targeted delivery of the drug. The size of nanoparticles falls in the range of 1 to 100 nm so as to permeate the BBB [140,141].

They are formulated in such way that makes them nontoxic, biodegradable and target-specific in nature. These types of nanosystems may effectively hold and distribute drugs and other neuroprotective molecules to the brain in the sense of treating AD [142,143,144]. The intranasal route plays a role in overcoming the BBB and targeting the drugs directly to the brain [145,146,147,148,149]. However, in order to optimize pharmacotherapy in patients with AD, nasal, dermal, and intravenous routes may be used to administer nanodevices to target the brain moving through BBB to improve bioavailability, pharmacodynamic properties and decrease the adverse effects of these medications [150,151,152]. The most common mechanisms of nanoparticle transportation include endocytosis, like receptor-mediated endocytosis, phagocytosis and pinocytosis, with receptor-mediated endocytosis being the most preferred method. The incorporated drug is delivered at the target site by diffusion and erosion or degradation processes. Some of the nanoparticles most often used are liposomes, polymeric nanoparticles, micro- and nanoemulsions and dendrimers.

### 3.1. Liposomes

These are bilayered phospholipids that are amphiphilic in nature (i.e., capable of transporting both hydrophilic and lipophilic drug molecules) [153,154]. Some antibodies have been proposed to inhibit the spread of Tau pathology by microglial phagocytosis of the antibody–Tau complex and to promote the clearance of lysosomal Tau in neurons after endosomal uptake [155,156]. The main components of liposomes include phosphatidyl choline, sphingomyelin and glycerophospholipids. Liposomes contain cholesterol that helps in maintaining its stability inside the serum. Their size ranges typically from 50 to 100 μm. The drug is encapsulated inside a lipid bubble which aids in its protection from degradation by enzymes and retains its effectiveness [157]. Liposomes have been studied by researchers extensively over the years.

### 3.2. Polymeric Nanoparticles

These are nanoparticles composed of synthetic or natural polymers, having their size in the range 1–100 nm [158]. The hydrophilic or hydrophobic nature of PNPs depends on the nature of the part forming its outermost layer. The mechanism of their transport to the target site can either be via receptor-mediated endocytosis or transcytosis of endothelial cells. The absorption of drugs can be enhanced by coating the PNPs with antibodies or PEG (polyethylene glycol), especially while delivering the drug via the intranasal route. Poly (n-butyl cyanoacrylate) loaded with the drug Rivastigmine showed improved drug delivery in case of AD when coated with polysorbate-80 [28]. In an experimental AD model, Aβ1-42 monoclonal antibody-decorated nanoparticle-based therapy against AD leads to complete correction of the memory defect [159]. With unique quantum properties that are promising to diagnostic and imaging purposes, nanoparticles can be prepared [160]. Micelles, nanogels, dendrimers and nanocapsules can be formulated as polymeric NPs [161,162].

### 3.3. Micro- and Nanoemulsions

These types of nanoparticles fall under the category of surfactant-based systems. The size of microemulsions ranges between 10 to 140 nm, while that of nanoemulsions lies around 100 nm [163]. These systems are also called oil-in-water (O/W) heterogeneous systems as they are formed by the dispersion of oil in water or any other aqueous medium. Hyaluronic acid-based nanoemulsion of curcumin and resveratrol used by Nasr showed promising results when delivered via the intranasal route to the brain. The preparation of the nanoemulsion was done using spontaneous emulsification method [139]. As a possible carrier of memantine for a direct nose-to-brain transmission, the produced nanoemulsion could be used [164].

### 3.4. Dendrimers

**Figure 4:**
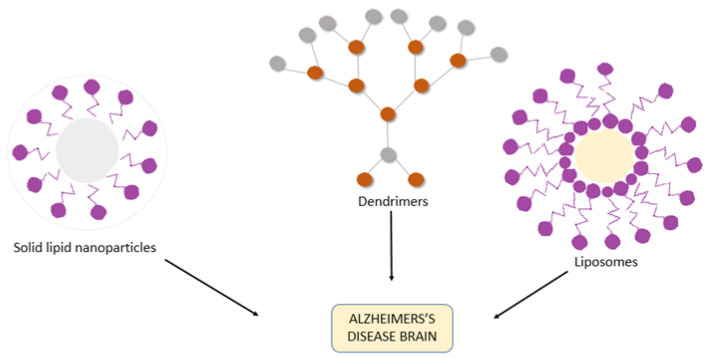
Diagrammatic representation of drug delivery options using nanotechnology for therapeutic purposes in AD.

These are polymeric branched, globular molecules also known as cascade molecules or arborols. They derive their name from their structural nature, which is to ramify progressively while originating from a core, similar to the behavior of the branches of a tree. Divergent and convergent are two methods of production of dendrimers. They offer a high drug loading capacity, including both the inner cavity and the outer surface of the dendrimer. The size of dendrimers can be easily regulated through careful selection of the monomers and the degree of polymerization. The only limitation is the issue of toxicity that is often faced when using these nanoparticles shown in Figure 4.

Polyamidoamines (PAMAMs), which are biocompatible, nonimmunogenic, and hydrophilic in nature, are the most used dendrimers in drug delivery. The nucleus of these dendrimers was composed of branching hydrophobic molecules of ethylenediamine and methylacrylate terminated by groups of carboxyl and amine. PAMAM dendrimers are used in drug delivery as carriers [165], diagnostic agents [166], gene transfection [167] and boron neutron capture treatment for metastatic brain tumors.

## 4. Conclusions

There are several hypothesis for the neurodegeneration process; however, the lack of availability of in vivo models makes the recapitulation of AD in humans impossible. Moreover, the drugs currently available in the market serve to alleviate the symptoms and there is no cure for the disease. There have been two major hurdles in the process of finding the same—the inefficiency in cracking the complexity of the disease pathogenesis and the inefficiency in delivery of drugs targeted for AD. This review discusses the different drugs that have been designed over the recent years and the drug delivery options in the field of nanotechnology that have been found most feasible in surpassing the blood–brain barrier (BBB) and reaching the brain.

## Figures and Tables

**Table 1:** Pharmacological treatments available for Alzheimer’s disease.

MOA	AChE Inhibitors			NMDA-Receptor Antagonist
Drug	Donepezil	Galantamine	Rivastigmine	Memantine
Indication	Moderate and severe AD	Mild and moderate AD	Mild and moderate AD	Moderate and severe AD
Initial dose	Tablet: 5 mg qd	Tablet/Oral soln.: 4 mg bid ER capsule: 8 mg qd	Capsule/Oral soln.: 1.5 mg bid Patch: 4.6 mg qd	Tablet/Oral soln.: 5 mg qd
Maximal dose	Tablet: 10 mg qd	Tablet/Oral soln.: 12 mg bid ER capsule: 24 mg qd	Capsule/Oral soln.: 6 mg bid Patch: 9.5 mg qd	Tablet/Oral soln.: 10 mg bid

ER: Extended release, MOA: Mechanism of action, AChE: Acetylcholine esterase, NMDA: N-methyl-D-aspartate. Source: [NIH Publication, 2008, https://www.uspharmacist.com/article/alzheimers-disease-increasing-numbers-but-no-cure].

## Data Availability

All data have been illustrated in the manuscript.
